# Integrating Statistical and Machine-Learning Approach for Meta-Analysis of Bisphenol A-Exposure Datasets Reveals Effects on Mouse Gene Expression within Pathways of Apoptosis and Cell Survival

**DOI:** 10.3390/ijms221910785

**Published:** 2021-10-05

**Authors:** Nina Lukashina, Michael J. Williams, Elena Kartysheva, Elizaveta Virko, Błażej Kudłak, Robert Fredriksson, Ola Spjuth, Helgi B. Schiöth

**Affiliations:** 1 Machine Learning Applications and Deep Learning Group, JetBrains Research, Kantemirovskaya Str., 2, 197342 St. Petersburg, Russia; elena.kartysheva@jetbrains.com (E.K.); virkoliza@gmail.com (E.V.); 2 Department of Neuroscience, Functional Pharmacology, University of Uppsala, BMC, Husargatan 3, Box 593, 751 24 Uppsala, Sweden; michael.williams@neuro.uu.se (M.J.W.); Helgi.Schioth@neuro.uu.se (H.B.S.); 3 Information Technologies and Programming Faculty, ITMO University, Kronverksky Pr. 49, bldg. A, 197101 St. Petersburg, Russia; 4 St. Petersburg School of Physics, Mathematics, and Computer Science, HSE University, 16 Soyuza Pechatnikov Street, 190121 St. Petersburg, Russia; 5 Department of Analytical Chemistry, Faculty of Chemistry, Gdańsk University of Technology, 11/12 Narutowicza Str., 80-233 Gdańsk, Poland; blazej.kudlak@pg.edu.pl; 6 Department of Pharmaceutical Biosciences, Molecular Neuropharmacology, Uppsala Biomedical Centre, University of Uppsala, Husargatan 3, Box 591, 751 24 Uppsala, Sweden; robert.fredriksson@farmbio.uu.se; 7 Department of Pharmaceutical Biosciences, Pharmaceutical Bioinformatics, Uppsala Biomedical Centre, University of Uppsala, Husargatan 3, Box 591, 751 24 Uppsala, Sweden; Ola.Spjuth@farmbio.uu.se; 8 Institute of Translational Medicine and Biotechnology, I. M. Sechenov First Moscow State Medical University, Trubetskay Str. 8, bldg 2, 119991 Moscow, Russia

**Keywords:** BPA, BPA-exposure datasets, DNA repair, cellular junction

## Abstract

Bisphenols are important environmental pollutants that are extensively studied due to different detrimental effects, while the molecular mechanisms behind these effects are less well understood. Like other environmental pollutants, bisphenols are being tested in various experimental models, creating large expression datasets found in open access storage. The meta-analysis of such datasets is, however, very complicated for various reasons. Here, we developed an integrating statistical and machine-learning model approach for the meta-analysis of bisphenol A (BPA) exposure datasets from different mouse tissues. We constructed three joint datasets following three different strategies for dataset integration: in particular, using all common genes from the datasets, uncorrelated, and not co-expressed genes, respectively. By applying machine learning methods to these datasets, we identified genes whose expression was significantly affected in all of the BPA microanalysis data tested; those involved in the regulation of cell survival include: *Tnfr2*, *Hgf-Met*, *Agtr1a*, *Bdkrb2*; signaling through *Mapk8* (*Jnk1*)); DNA repair (*Hgf-Met*, *Mgmt*); apoptosis (*Tmbim6*, *Bcl2*, *Apaf1*); and cellular junctions (*F11r*, *Cldnd1*, *Ctnd1* and *Yes1*). Our results highlight the benefit of combining existing datasets for the integrated analysis of a specific topic when individual datasets are limited in size.

## 1. Introduction

Bisphenols have been in commercial use as plasticizers for over 70 years. They are reported to be estrogenic mimics that may interfere with hormonal homeostasis. One very prevalent bisphenol, bisphenol A (BPA), is used for manufacturing polysulfones and polycarbonate plastics, epoxy resins, and thermal paper. BPA is considered an endocrine and metabolic disruptor, able to interfere with important physiological systems, such as insulin-glucagon signaling [1,2,3,4]. Comparatively, BPA has one of the highest production volumes of any chemical worldwide, with global production estimated at 7.7 million metric tons in 2015, and it is expected to reach 10.6 million metric tons by 2022 [5]. Mammals are exposed to BPA daily through several routes, such as the consumption of food and drink, drugs, air born inhalation, and contact materials, such as various plastics, medical devices, and store receipts [6,7,8]. However, the main exposure route of BPA is through diet as many food packages contain BPA, allowing it to leach into the food and be ingested [6,9,10,11,12]. Due to its pervasiveness in the environment, BPA has been detected in the urine and sera in 90% of the people sampled, as well as in the amniotic fluid, placenta, and breast milk of women [7,13,14,15,16,17,18]. It has become increasingly clear that BPA can bioaccumulate in the food chain. In fact, in a study in Africa, BPA reached very high concentrations in food (940 ng/g), biological fluids (209 ng/mL), consumer and PCPs (3.6 μg/g), and semisolids (154 μg/g) [19].

Considering the prevalence of BPA in the biome and its suspected disruption of human physiology, many groups have used various model organisms, including mice or human cell lines, in an attempt to determine how BPA interacts with different biological signaling systems [20]. Some of these groups have performed a microarray analysis after BPA exposure and deposited this information in public data banks [21,22]. However, most of the published datasets are relatively small, and meta-analysis studies that attempt to integrate existing microarray datasets regarding exposure to BPA are currently lacking. There is an opportunity to combine the existing datasets to improve the accuracy of the identified genes and pathways involved in BPA exposure.

It is somewhat surprising that most of the literature on data mining and chemometric data calculations refers to the exposure-instrumental/biological testing loop while less attention has been paid to exposure-gene expression correlations treatment with advanced environmetrics. The impact of BPA on cardiometabolic factors [23] has shown a positive correlation between patients’ BPA concentrations and diabetes (87%), overweight (28%), obesity (85%), elevated waist circumference (100%), cardiovascular diseases (80%), and hypertension (66%) in cross-sectional studies. Unfortunately, none of these studies can confirm if BPA can be proven as a risk factor of the observed anomalies or if these elevated BPA concentrations result from the already pre-disordered status of the given patient. The problem is of increasing importance as BPA (at environmentally relevant concentrations) has been confirmed to affect pre-implanted embryos and has been detected in samples of serum and follicular fluid collected from women and the umbilical cord at ca. 1–2 ng/mL levels [11]. For this reason, it is warranted to pay more attention to studies on the exposure-gene expression loop to unveil these interrelationships.

The amount of functional genomics data in the form of expression profiles from various experimental designs and model organisms are increasing rapidly, with over 1000 new submissions yearly to the ArrayExpress repository (https://www.ebi.ac.uk/arrayexpress/, accessed on 10 February 2020) [24]. Currently, the largest repositories of public functional genomics data are ArrayExpresses and NCBI Geo (https://www.ncbi.nlm.nih.gov/geo/, accessed on 10 February 2020) [25], which, in January 2020, contained 72,578 and 97,273 unique experiments, respectively; although 59,374 were found in both databases and, hence, redundant [26]. Currently, the majority of these are in the form of microarray data, although, since 2018, the number of RNASeq experiments submitted to ArrayExpress is higher than the number of microarray submissions [24]. Utilizing these databanks for novel large-scale analysis poses challenges due to the diversity of the technical platforms used to generate the data, resulting in differences in file formats, signal levels, and data variance, as well as differences in experimental design. Although several attempts have been made to simplify data retrieval and data selection, such as the All Of gene Expression (AOE) web portal [26] and Biostudies database, which is now becoming the successor of ArrayExpress [24], the challenges of between-experiments normalization and adjusting for the differences in experimental design remain. These difficulties in combining and analysing functional genomics data from various sources necessitate innovative and more powerful methods to utilize these data for novel analyses.

In this manuscript, we studied the gene expression changes from four available microarray datasets of mice under the influence of BPA exposure. The standard approach in analysing gene expression changes is to perform differential expression analysis with statistical tests for differences in intensity [27]. We performed this traditional differential gene expression analysis of individual GEO datasets. However, this method suffers from various issues, such as uncertainty in *p*-value choice to select the right set of “important” genes in terms of biological effects and the necessity of dealing with the problem of multiple comparisons. In contrast, machine learning methods, especially feature selection methods, are widely used today in gene expression analysis, providing the ability to select the right set of “important” genes in terms of the quality of the prediction model [27,28]. In this study, we focused on applying machine learning methods in terms of feature selection (FS), revealing key genes influenced by BPA exposure.

We constructed three joint datasets following three different correlation-based pre-processing approaches, namely using all of the common genes through four GEO datasets, uncorrelated, and no co-expressed genes, respectively. By applying machine learning methods to these joint datasets, we identified genes whose expression was significantly changed in all of the BPA microanalysis data tested. We went on to determine that a subset of these genes is involved in the regulation of cell survival and apoptosis. Our results highlight the benefit of combining existing datasets for integrated analysis for a specific topic when individual datasets are limited in size, in our case when studying the effects of BPA.

## 2. Results

### 2.1. Differential Gene Expression Analysis

**Figure 1:**
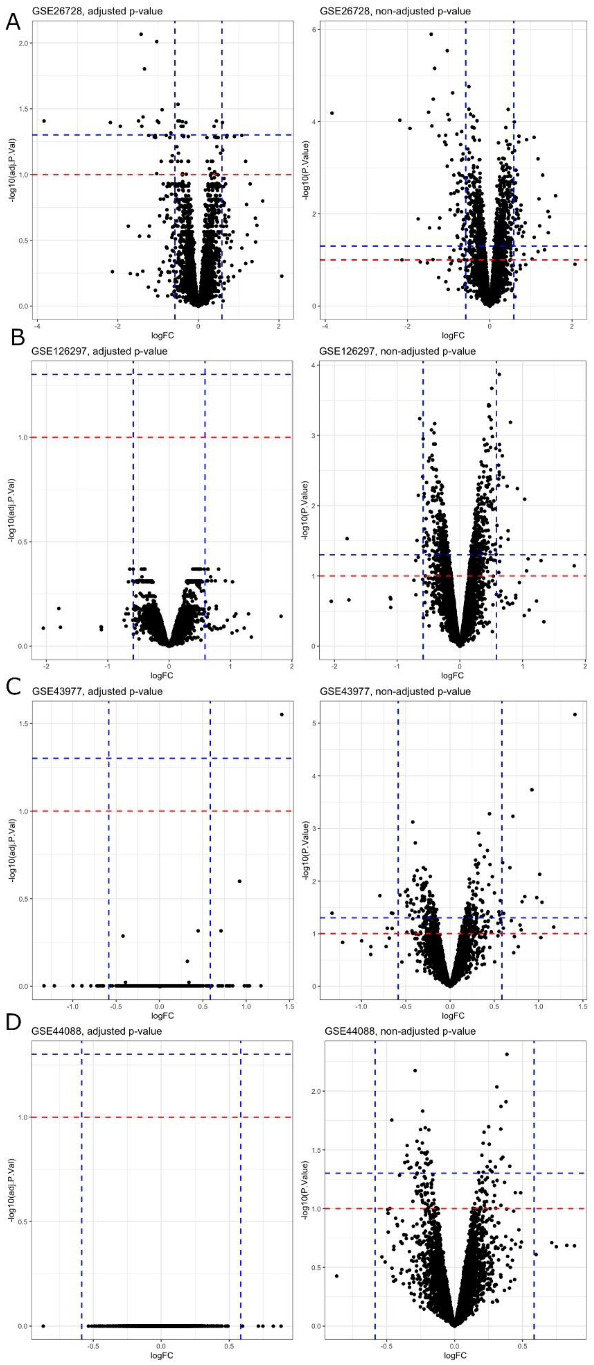
Volcano plots of differential expression analyses, using adjusted *p*-values (left column) and non-adjusted *p*-values (right column), for (**A**) GSE26728, (**B**) GSE126297, (**C**) GSE43977, and (**D**) GSE44088 datasets. Dashed blue lines are used to designate *p*-value of 0.05, dashed red lines for *p*-value of 0.1. Only GSE26728 has differentially expressed genes with respect to both adjusted and non-adjusted *p*-values. Other datasets have differentially expressed genes with respect to non-adjusted *p*-values only.

Differential gene expression analysis was performed in several ways in terms of statistical significance. As described in the Methods section, we declared a gene differentially expressed if an observed expression difference between two experimental conditions reported an adjusted *p*-value < 0.05. We also performed the same analysis with an adjusted *p*-value < 0.1, non-adjusted *p*-value < 0.05, and non-adjusted *p*-value < 0.1 (Figure 1). After applying multiple adjustment corrections, the analysis determined that GSE26728 was the only dataset with differentially expressed genes. All of the other datasets examined did not show any differentially expressed genes, neither with an adjusted *p*-value < 0.05 nor with an adjusted *p*-value < 0.1. On the contrary, all the datasets showed differentially expressed genes with both a non-adjusted *p*-value < 0.05 and a non-adjusted *p*-value < 0.1. Therefore, we could state that there were no common differentially expressed genes among the four datasets.

### 2.2. Machine Learning Methods

In our study, we found that ensemble-based methods (Section 4.2.1) tended to overfit the data studied (Table 1). Both the Random Forest (RF) model and the Support Vector Machine (SVM) ensemble model were able to learn the training dataset, producing 1.0 training accuracy, but failed to generalize, producing a test accuracy only slightly higher than 0.5. Due to the high differences in training and test accuracies for fitted models, we did not use feature sets from these models in any subsequent analysis.

In contrast, the iterative model seemed to be able to construct more meaningful feature sets before it overfit our data. The iterative feature selection procedure (Section 4.2.2) with two binary classification models, Naïve Bayesian classifier (NB) and Logistic Regression (LR), were applied to the datasets. The resulting feature sets, composed of selected genes, were used to train a single SVM model in order to prove the predictive ability of the selected features (Section 4.3). Table 2 and Table 3 show the test/training cross-validation accuracies and ROC AUC scores of the SVM model. Although the training accuracies and ROC AUC scores remained close to 1.0, the differences between the training and test scores significantly decreased, showing the ability of the models to generalize.

### 2.3. Gene Lists Analysis

**Figure 2:**
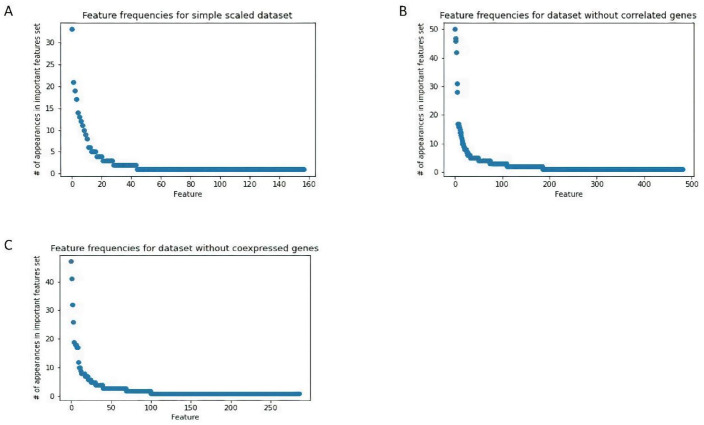
Feature frequencies, obtained by 100 runs of iterative feature selection procedure, for (**A**) simple scaled dataset, (**B**) dataset without correlated genes and, (**C**) dataset without co-expressed genes. There are 4 features for the simple scaled dataset, 6 features for the dataset without correlated genes, and 7 features for dataset without co-expressed genes, which have noticeably higher frequencies than other features.

In the next step, we analyzed the number of appearances of each feature in the feature sets, obtained by 100 runs of the iterative feature selection procedure on each of the datasets (ref. to Section 4.4.1). We considered the most frequent features to be the most important genes in terms of distinguishing between the BPA-exposed and control samples. For these genes, pathway analysis was performed using DAVID [29] to determine the most enriched pathways and biological processes within each dataset (Section 4.4.2). This revealed that the most frequent genes from the simple scaled dataset (Figure 2A and Table 4), without correlated genes dataset (Figure 2B and Table 5), and without co-expressed genes dataset (Figure 2C and Table 6) did not cluster together in any Gene Ontology (GO) biological processes (BP). By examining the top genes for all of the datasets, we could observe 24 common genes (Table 7).

Next, the 24 common genes were used for pathway analysis using DAVID to find the most enriched biological processes. The functional annotation in DAVID showed that one cluster of genes (*Dbp*, *P2ry1*, *Tbl1x*, *Nrip1*, and *Yes1*) was related to GO:004594: the positive regulation of transcription from RNA polymerase II promoter. There were also two genes belonging to the ephrin receptor signaling pathway (GO:0048013), *Efnb2* and *Efna4*.

We then examined important features for the intersection of the top 30 genes from the machine learning models with the cut-off of 20 appearances. It was expected that important (the most frequent) features for datasets without correlated and without co-expressed genes would be similar due to the similarity of the pre-processing procedure. Five common genes among the top 30 genes were found for these datasets: *F11r*, *Pfkfb1*, *Zfp839*, *Csn1s2b*, *Yes1*. Moreover, there were two genes (*Rad9a*, *Senp5*) that appear in the top 30 genes in the simple scaled dataset only.

**Figure 3:**
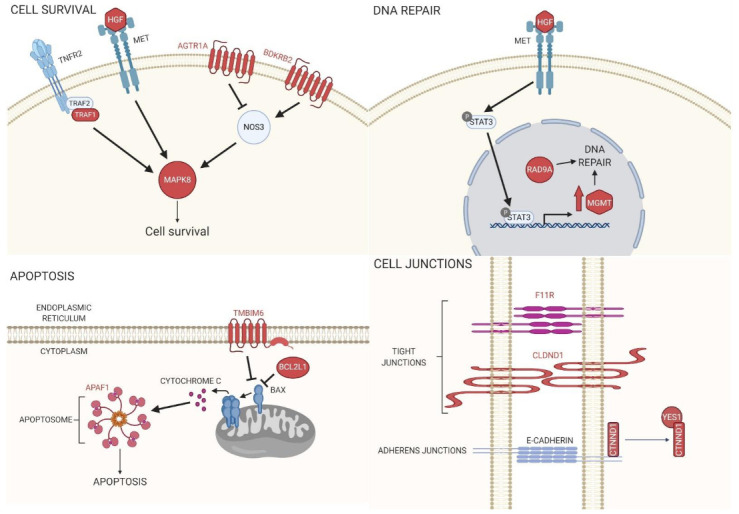
Biological pathways for the top 30 genes (Names or proteins in red = top 30 genes). A subset of the genes recovered in our analysis relates to the regulation of cell survival, DNA repair, apoptosis, and cellular junctions. In fact, many of the pathways recovered, including *Tnfr2*, *Hgf-Met*, *Agtr1a*, *Bdkrb2*, signal through *Mapk8* (also known as *Jnk1*) to regulate cell survival. One of these pathways, *Hgf-Met*, also functions to regulate another recovered gene, *Mgmt*, to allow for DNA repair. Two of the recovered genes, *Tmbim6* and *Bcl2L*, inhibit *Bax* in order to prevent apoptosis, while one gene, *Apaf1*, is necessary for forming apoptosomes to induce apoptosis. Cellular junctions are also centrally important for cell survival, and four of the genes recovered, *F11r*, *Cldnd1*, *Ctnd1*, and *Yes1*, function for maintain cellular junctions.

The genes from the top 30 important genes dataset were also utilized in the pathway analysis using DAVID to determine the most enriched biological processes. Although most of the genes did not form any obvious clusters, the functional annotation in DAVID showed that the two largest clusters of Gene Ontology (GO) biological processes (BP) were related to the regulation of apoptosis (GO:0042981) and proteolysis (Table 8). In fact, two clusters, having some gene overlap, were related to the general regulation of apoptosis or the negative regulation of apoptosis (GO:0043066) (Table 8 and Figure 3).

## 3. Discussion

Bisphenols are important pollutants that significantly infiltrated the biome. Their potential to disrupt physiology has led several groups to perform microarray analysis using biological material from mice after BPA intervention. The large datasets created by these works have been deposited in public data banks [21,22], but no other analysis has been performed. Using machine learning, we mined a subset of these microarray datasets and were able to define not only a method for performing a meta-analysis of these large datasets but also produce pathways conserved across different BPA interventions within a species.

Our research confirms the importance of combining datasets in a meta-analysis but also highlights the importance of different pre-processing steps before applying machine learning methods, especially for small datasets. In this study, we focused on various correlation-based gene averaging processes. We showed that the usage of these strategies leads to different, but, in general, related, solutions. This suggests that co-expression-based pre-processing produces a dataset modification that promotes a solution candidate. Using hard voting, the final result was aggregated from the results of running three models on dataset modifications. The strategies to improve this process might include a deeper investigation of the differences in outcomes between the models, as well as more sensitive aggregation.

BPA exposure has been shown to disrupt mitochondria integrity, leading to elevated ROS levels and apoptosis rates in human granulosa and HT-22 cells, which are derived from the mouse brain [30,31]. The BPA-inhibited proliferation of neural progenitor cells and rat embryonic midbrain cells through the suppression of the JNK signaling pathway has also been reported [32,33]. Our gene ontology analysis using DAVID indicated that a significant group of the top 30 transcripts recovered from machine learning models were linked to the regulation of apoptosis (see Table 8). Furthermore, by using DAVID and STRING, it became evident that many of the genes encoding these transcripts categorized as being involved in the regulation of apoptosis were, in fact, cell survival pathways that converged on Mitogen-Activated Protein Kinase 8 (Mapk8, also known as Jnk1) (Figure 3). The protein of one of the transcripts recovered, Hepatocyte growth factor (Hgf), activates MET Proto-Oncogene, Receptor Tyrosine Kinase (Met), which not only regulates Mapk8 activation but also leads to increased transcriptional expression of another gene in our dataset, *O-6-Methylguanine-DNA Methyltransferase* (*Mgmt*) [34]. Mgmt and Rad9a proteins, whose transcript levels were also shown to be affected by BPA, are both involved in repairing damaged DNA [35]. Furthermore, in mouse macrophages, it was determined that BPA-induced mitochondrial disruption reduced BCL2 protein expression, which led to caspase-dependent apoptosis [36]. In our study, one of the top 30 genes was *Bcl2l1*, whose protein is known to inhibit Bax-induced apoptosis (Figure 3). Furthermore, the protein of another gene recovered in the study, *Apaf1*, acts downstream of Bax, and, along with Caspase-9, forms an apoptosome to induce apoptosis (Figure 3) [37]. Interestingly, we also showed that *Tmbim6* transcript levels are affected by BPA, and *Tmbim6* was shown to inhibit Bax-induced apoptosis (Figure 3) [38].

Three of the top 30 genes, *F11 Receptor* (*F11r*, also known as *Jam-1*), *Claudin Domain Containing 1* (*Cldnd1*), and *Catenin Delta 1* (*Ctnnd1*), are associated with either tight or adherens junctions (see Table 8 and Figure 3). In a recent study, the reproductive toxicity of BPA was investigated. Male CD-1 mice were orally administrated BPA, and the results showed that this exposure was sufficient to induce disorders in spermatogenesis, including damaging the tight junctions between Sertoli cells [39]. Another study examined the effect of BPA in female rats on the expression levels of tight junction (TJ) transcripts in the uterus during early pregnancy. This study found profound alterations in the TJ gene transcript levels of uterine epithelial cells when the rats were exposed to BPA, which led to changes in fluid and ion transport across the epithelium, blocking the receptivity of the uterus to blastocyst implantation [40]. In fact, this study saw profound effects on claudin transcript levels, such as *Cldnd1*, including low expression levels or even the loss of expression.

Interestingly, our study recovered transcripts for two receptors that are in pathways regulated by Angiotensin I Converting Enzyme (ACE), *Angiotensin II Receptor Type 1a* (*Agtr1a*), and *Bradykinin Receptor B2* (*Bdkrb2*) (see Table 8 and Figure 3). In both rat cardiac cells and human endothelial cell lines, it was shown that BPA was proangiogenic, including the upregulation of Nitric Oxide Synthase 3 [41,42,43]. In another report, it was discovered in rat striatum that the inhibition of ACE was able to alleviate the ROS-inducing effects of a BPA + 1-methyl-4-phenylpyridinium ion (MPP(+)) mixture [44]. Interestingly, both *Agtr1a* and *Bdkrb2* signal upstream of *Nos3*, where *Agtr1a* leads to *Nos3* inhibition and *Bdkrb2* leads to activation (Figure 3).

In terms of computational methods, in this paper, we suggest using a new cross-validation-based greedy feature selection algorithm with three different preprocessing strategies. Using this approach, one has the flexibility to incorporate different machine learning models and stopping criteria into the feature selection procedure depending on the properties of the data. We also provided gene importance analysis based on the frequencies of the genes’ appearances in the feature lists from 100 runs of the proposed algorithm. For small datasets, this process is more stable than using feature selection techniques based on a single run.

Our results highlight the value of integrating data from multiple datasets for co-analysis, revealing new biological knowledge. However, a key limitation of our study is still a lack of publicly available microarray data after BPA exposure, which restricts our investigation to the baseline machine learning methods. This is also an important constraint for analyzing the differences between the results from datasets without correlated and without co-expressed genes. We used co-expression analysis with the WGCNA package for each GEO dataset, but it should be carefully used for datasets with less than 15 samples [45]. This means that a pre-processing method should be attentively chosen based on the available data.

In summary, we developed a new approach for the meta-analyses of microarray data, which could be very useful for analyzing other datasets relating to any environmental pollutants. The pathways that we have identified align well with the previous evidence for the molecular actions of BPA and prompt further studies into pathways that relate to the regulation of cell survival, DNA repair, apoptosis, and cellular junctions.

## 4. Materials and Methods

### 4.1. Dataset Collection of BPA-Exposure-Related Data

We restricted our survey to the datasets devoted to BPA-exposure experiments using male mice. Four publicly available microarray datasets from the GEO database were examined: GSE26728 [21], GSE126297 [22], GSE43977 [43], and GSE44088 [43]. In GSE26728, liver gene expression was measured from CD-1 mice exposed for 28 days to bisphenol A at doses 0 (controls), 50 (TDI or low dose), or 5000 µg/kg/day (NOAEL or high dose) via food spiking [21]. The GSE126297 dataset used pancreatic islets from OF1 male mice after exposure of organisms to 100 μg/kg/day (two injections of 50 μg/kg/day) for four days [22]. The GSE43977 and GSE44088 datasets used hepatic samples from C57BL/6J mice [43] after exposure to ~21.93 mM (5000 ppm) for 7 days and 10 μM for 24 h, respectively. Four datasets have 41 samples in total, 21 control untreated samples and 20 treated samples.

We examined each dataset separately for differential expression analysis. For ML-based analysis, we combined datasets following three different strategies. Below is the detailed description of all pre-processing procedures.

#### 4.1.1. Data Pre-Processing for Differential Expression Analysis of Individual Datasets

In the bioinformatic pipeline, we examined each dataset separately, where datasets themselves were given log2-transformed values. Expression data files were pre-processed using the R *limma* package (version 3.42.0) [46]. We annotated datasets with Entrez ID and dropped NA values. We defined low-expression genes with a constant threshold for log-transformed probe intensity values and removed them manually from the dataset [47]. We also removed probe replicates using the *avereps* function and performed quantile normalization using the *normalizeBetweenArrays* function.

#### 4.1.2. Data Pre-Processing for Machine Learning-Based Analysis for Combined Datasets

In order to analyze combined datasets, we reduced each dataset to the common genes set among all datasets. This left us with four datasets having 6742 genes in each. Then, we scaled intensity values for each gene in each dataset in the range of 0 to 1, following Equation (1).

(1) $$x_{scaled}=\frac{x-min\left(x\right)}{max\left(x\right)-min\left(x\right)},$$

where x is an intensity value for the specific gene.

Finally, we combined scaled datasets into a single dataset, following three different strategies. The first strategy was not to use any modification. The second and third strategies use two different ways to construct independent feature sets in order to meet the requirement of machine learning algorithms with independence assumptions between the features.

Simple scaled dataset. The first strategy is to combine four datasets without any modifications, resulting in a dataset with a matrix size of 41×6742.

Dataset without correlated genes. In the second strategy, we built a correlation graph. In this graph, vertices correspond to the genes, and edges correspond to the correlated genes with α level of Pearson correlation. Then, we replaced each connectivity component with an averaged value of its vertices. Thus, the new dataset consists of uncorrelated elements, representing genes or averaged groups of genes. We varied α from 0.7 to 0.99 and finally used 0.7 because, for higher levels, most of the genes did not belong to any correlation cluster. This strategy resulted in a dataset with a shape of 41x5704.

**Figure 4:**
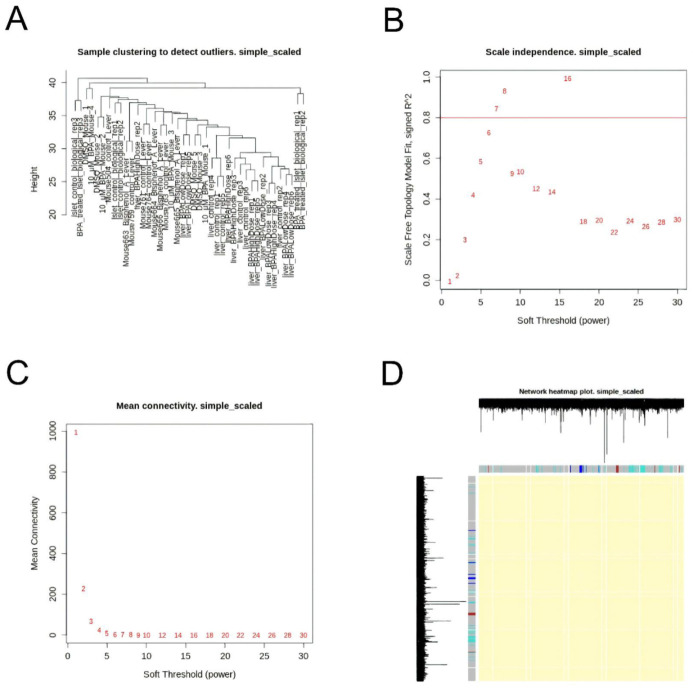
(**A**) Sample tree for combined dataset of GSE26728, GSE126297, GSE43977, GSE44088. Scale independence (**B**) and Mean connectivity (**C**) for combined dataset of GSE26728, GSE126297, GSE43977, GSE44088. Soft threshold is the lowest power for which the scale-free topology fit index curve flattens out upon reaching a high value. (**D**) Genes heatmap for combined dataset of GSE26728, GSE126297, GSE43977, GSE44088.

Dataset without co-expressed genes. In the third strategy, we used the R package WGCNA (version 1.46) [48] to build co-expressing clustering based on biweight midcorrelation. For a combined scaled dataset, we analyzed genes’ co-expression with the following steps. First, we clustered the samples (in contrast to clustering genes that will be described later) with *hclust* function to see if there are any potential outliers. Figure 4A shows a sample tree without any outliers.

Then, we built a gene-gene similarity network with soft-threshold power selection using *pickSoftThreshold* function. Figure 4B,C show soft-threshold power selection. We chose the threshold equal to 7 (this value is the lowest power for which the scale-free topology fit index curve flattens out upon reaching a high value).

In the next step, we built the corresponding gene network and identified modules within each network. Figure 4D shows the heatmap for the gene network. Each row and column of the heatmap corresponds to a single gene. The heatmap can depict adjacencies or topological overlaps, with light colors denoting low adjacency (overlap) and darker colors higher adjacency (overlap). In addition, the gene dendrograms and module colors are plotted along the top and left side of the heatmap. Based on results presented in Figure 4D, one can conclude that genes taken into account do not have strong co-expression.

Finally, we averaged genes among each cluster. In total, 3 clusters with 1094 genes were averaged. This strategy resulted in a dataset with a matrix size of 41×5651. As a result, we have obtained the simple combined dataset, the dataset without correlated genes, and the dataset without co-expressed genes.

#### 4.1.3. Differential Gene Expression Analysis

We performed differential expression analysis using the R package *limma* (version 3.42.0) [46]. Benjamini–Hochberg correction was applied as multiple testing correction. A gene was declared differentially expressed if an observed expression difference between two experimental conditions was equal or more than 1.5 and statistically significant (adjusted *p*-value < 0.05).

### 4.2. Machine Learning-Based Genes Selection

We used machine learning to build binary classification models considering genes as features and then find “important” features in terms of distinguishing BPA-exposed samples from control ones. In particular, we used two different machine learning-based approaches, namely ensemble-based methods and new iterative feature selection procedure.

#### 4.2.1. Ensemble-Based Approach

We used Random Forest and Support Vector Machine (SVM) ensemble methods, with feature bagging, to all three datasets. Random Forest is a widely used classification model; we used it with 1000 Gini impurity-based trees and 100 features in each tree. In order to find the most “important” genes, we used Gini importance, which is computed as the total reduction of Gini impurity brought by that feature. Similar to Random Forest, we built an SVM ensemble with feature bagging. As a feature importance criterion, we used weights, assigned to each feature by the SVM classifier.

#### 4.2.2. Iterative Feature Selection Procedure

**Figure 5:**
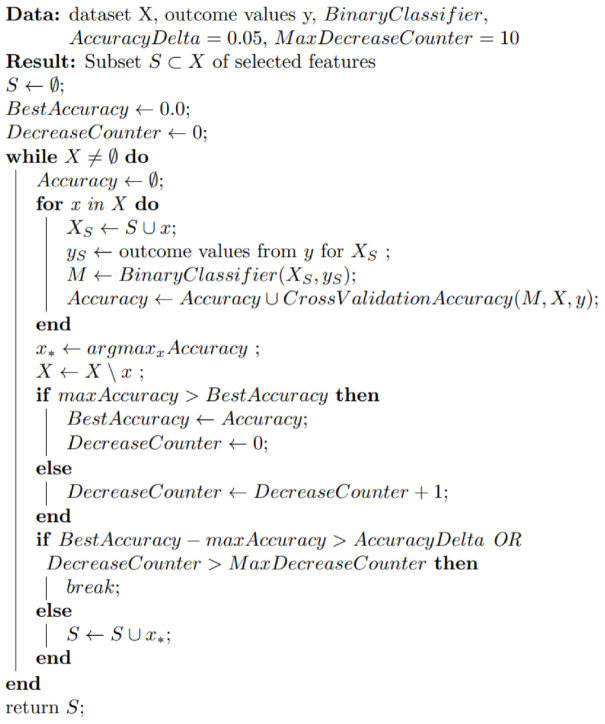
The algorithm of the cross-validation-based greedy selection procedure. The algorithm takes as inputs the following parameters: dataset X (gene features of each of three datasets, simple scaled, without correlated genes, and without co-expressed), BinaryClassifier (a function of binary classification), AccuracyDelta (the minimum significant difference in the accuracy score), and MaxDecreaseCounter (the maximum number of steps to evaluate in case of accuracy decrease). The iterative feature selection procedure returns a subset of selected features.

We constructed a cross-validation-based greedy feature selection procedure (Figure 5). On each step, this procedure tries to expand a feature set by adding a new feature. It fits a model with different alternatives and selects a feature that is the best in terms of cross-validation accuracy on that step.

An alternative to this idea could be a Recursive Feature Elimination procedure (RFE), which fits a model once and iteratively removes the weakest feature until the specified number of features is reached. The reason why we did not use RFE procedure is its inability to control the fitting process, while our greedy selection algorithm provides us an opportunity to set up useful stopping criteria. We stopped when there was no significant increase in cross-validation accuracy, which helped us overcome overfitting.

Because of the small number of samples in our dataset, we used 50/50 split in cross-validation. This led to an issue of unstable feature selection at each step. In order to reduce this instability, we ran the procedure 100 times and calculated a gene’s appearances in “important genes” lists.

The crucial step of the algorithm is to train a binary classifier, which could be any appropriate classification model. In our study, we focused on strong baseline models. We used Logistic Regression with L1 and L2 penalties for the simple combined dataset and Naive Bayesian classifier for the datasets without correlated or co-expressed genes. Naive Bayesian classifier is known to be a strong baseline for problems with independence assumptions between the features. It assigns a class label y_NB from possible classes Y following maximum a posteriori principle (Equation (2)):

(2) $$y_{NB}=argmax_{y\in Y}P\left(y\right)\prod^{}{}_{i}P\left(x_{i}\vee y\right),$$

under the “naive” assumption that all features are mutually independent (Equation (3)):

(3) $$P\left(x_{1},x_{2},\ldots ,x_{n}\vee y\right)=P\left(x_{1}\vee y\right)P\left(x_{2}\vee y\right)\ldots P\left(x_{n}\vee y\right),$$

where *x_i_* stands for an intensity value for the specific gene *i*, *y* stands for a class label, $P\left(x_{i}\vee y\right)$ stands for a probability of class y for the intensity value *x_i_*, *P(y)* stands for *y* class probability. Both probabilities $P\left(x_{i}\vee y\right)$ and *P(y)* are estimated with relative frequencies in the training set.

Logistic Regression is a simple model that assigns class probabilities with sigmoid function of linear combination (Equation (4)):

(4) $$y_{LR}=argmax_{y\in Y}\sigma \left(yw^{T}x\right),$$

where *x* stands for a vector of all intensity values, *w* stands for a vector of linear coefficients, *y* stands for a class label and σ is a sigmoid function.

We used it with ElasticNet regularization, which includes penalties to L1 and L2 norms of weight vector w.

### 4.3. Genes Selection Validation

In order to prove predictive ability of selected features, we used them in the S classifier, which is known to be a strong model for binary classification. We checked the increase in cross-validation ROC AUC scores for each feature set.

### 4.4. Gene Lists Analysis

#### 4.4.1. Identification of the Most Important Genes

We calculated the genes’ appearances in feature lists from 100 runs of the algorithm (Figure 5). From these frequencies, we were able to range genes in each dataset in terms of their importance for binary classification.

In order to compare gene lists to each other, we built a summary table using the top 30 genes of each dataset. We also annotated them with corresponding *p*-values from differential expression analysis.

#### 4.4.2. Annotation and Pathway Analysis

Pathway enrichment analysis was performed in DAVID (Database for Annotation, Visualization and Integrated Discovery) and PANTHER, using Gene Ontology (GO), and Reactome databases (PMID: 22543366; PMID: 30804569; PMID: 31691815). The MetaCore default setting of false discovery rate (FDR) < 0.05 was used as threshold for significance in enrichment analysis.

## Figures and Tables

**Table 1:** Test/train cross-validation accuracy for ensemble models. Random Forest and SVM ensemble models were applied to simple scaled (simple_scaled), without correlated genes (without_correlated), and without co-expressed genes (without_coexpressed) datasets. Both Random Forest and SVM ensemble models failed to generalize on each of the datasets.

Model	Simple_Scaled	Without_Correlated	Without_CoexPressed
Random Forest	0.54/1.0	0.53/1.0	0.54/0.94
SVM ensemble	0.52/1.0	0.53/1.0	0.54/1.0

**Table 2:** Test/train cross-validation accuracies of SVM model, trained on all genes and genes selected by the iterative feature selection procedure with Naïve Bayesian classifier or Logistic Regression classifier. SVM model was applied to simple scaled (simple_scaled), without correlated genes (without_correlated), and without co-expressed genes (without_coexpressed) datasets. In contrast to all genes as a feature set, genes selected by the iterative procedure show predictive ability.

	Simple_Scaled	Without_Correlated	Without_CoexPressed
All genes	0.54/1.0	0.55/1.0	0.54/1.0
Top genes from Naive Bayesian classifier	-	0.82/0.9	0.74/0.84
Top genes from Logistic Regression	0.6/0.73	-	-

**Table 3:** Test/train cross-validation ROC AUC scores of SVM model, trained on all genes and genes selected by the iterative feature selection procedure with Naïve Bayesian classifier or Logistic Regression classifier. SVM model was applied to simple scaled (simple_scaled), without correlated genes (without_correlated), and without co-expressed genes (without_coexpressed) datasets. In contrast to all genes as a feature set, genes selected by the iterative procedure show predictive ability.

	Simple_Scaled	Without_Correlated	Without_CoexPressed
All genes	0.62/1.0	0.60/1.0	0.62/1.0
Top genes from Naive Bayesian classifier	-	0.93/0.97	0.85/0.93
Top genes from Logistic Regression	0.72/0.83	-	-

**Table 4:** The most frequent genes within 100 runs of the iterative feature selection procedure on the simple scaled dataset.

Entrez ID	Gene Symbol	Gene Name	Frequency/100
654810	*Appbp2os*	*Amyloid beta precursor protein (cytoplasmic tail) binding protein 2, opposite strand*	33
110213	*Tmbim6*	*Transmembrane BAX inhibitor motif containing 6*	21
22121	*Rpl13a*	*Ribosomal protein L13A*	19
11603	*Agrn*	*Agrin*	17

**Table 5:** The most frequent genes within 100 runs of the iterative feature selection procedure on the dataset without correlated genes.

Entrez ID	Gene Symbol	Gene Name	Frequency/100
12984	*Csf2rb2*	*Colony stimulating factor 2 receptor, beta 2, low-affinity (granulocyte-macrophage)*	50
19367	*Rad9a*	*RAD9 checkpoint clamp component A*	47
230085	*Phf24*	*PHD finger protein 24*	46
15213	*Hey1*	*Hairy/enhancer-of-split related with YRPW motif 1*	42
72805	*Zfp839*	*Zinc finger protein 839*	31
229279	*Hnrnpa3*	*Heterogeneous nuclear ribonucleoprotein A3*	28

**Table 6:** The most frequent genes within 100 runs of the iterative feature selection procedure on the dataset without co-expressed.

Entrez ID	Gene Symbol	Gene Name	Frequency/100
12984	*Csf2rb2*	*Colony stimulating factor 2 receptor, beta 2, low-affinity (granulocyte-macrophage)*	42
15213	*Hey1*	*Hairy/enhancer-of-split related with YRPW motif 1*	41
230085	*Phf24*	*PHD finger protein 24*	34
19367	*Rad9a*	*RAD9 checkpoint clamp component A*	31
72805	*Zfp839*	*Zinc finger protein 839*	26
229279	*Hnrnpa3*	*Heterogeneous nuclear ribonucleoprotein A3*	25
12593	*Cdyl*	*Chromodomain protein, Y chromosome-like*	20

**Table 7:** Genes common among all important features from three datasets: simple scaled dataset, dataset without correlated genes and dataset without co-expressed genes.

Entrez ID	Gene Symbol	Gene Name	GO Terms	Biological Process
12984	*Csf2rb2*	*colony stimulating factor 2 receptor, beta 2, low-affinity (granulocyte-macrophage)*	PC00197 ^1^	Transmembrane signal receptor
19367	*Rad9a*	*RAD9 checkpoint clamp component A*	GO:0000076	DNA replication checkpoint
18441	*P2ry1*	*purinergic receptor P2Y, G-protein coupled 1*	GO:0071407 ^2^	cellular response to organic cyclic compound
320213	*Senp5*	*SUMO/sentrin specific peptidase 5*	GO:0070646	Protein modification by small protein removal
22612	*Yes1*	*YES proto-oncogene 1, Src family tyrosine kinase*	GO:0008283	Cell population proliferation
268903	*Nrip1*	*nuclear receptor interacting protein 1*	GO:0071392	cellular response to estradiol stimulus
13642	*Efnb2*	*ephrin B2*	GO:0007411	Axon guidance
21372	*Tbl1x*	*transducin (beta)-like 1 X-linked*	GO:0016575	Histone deacetylation
56530	*Cnpy2*	*canopy FGF signaling regulator 2*	GO:0010988 ^2^	Regulation of low-density lipoprotein particle clearance
13885	*Esd*	*esterase D/formylglutathione hydrolase*	GO:0016788	Hydrolase activity, acting on ester bonds
13639	*Efna4*	*ephrin A4*	GO:0007411	Axon guidance
11607	*Agtr1a*	*angiotensin II receptor, type 1a*	GO:0006954	Inflammatory response
116837	*Rims1*	*regulating synaptic membrane exocytosis 1*	GO:0046928	Regulation of neurotransmitter secretion
654810	*Appbp2os*	*amyloid beta precursor protein (cytoplasmic tail) binding protein 2, opposite strand*	GO:0008017	Microtubule binding ^2^
213773	*Tbl3*	*transducin (beta)-like 3*	GO:0000462	Maturation of SSU-rRNA from tricistronic rRNA transcript
13170	*Dbp*	*D site albumin promoter binding protein*	GO:0000977	RNA polymerase II regulatory region sequence-specific DNA binding
140475	*Bsnd*	*barttin CLCNK type accessory beta subunit*	GO:0006821	Chloride transport
13046	*Celf1*	*CUGBP, Elav-like family member 1*	GO:0000380	Alternative mRNA splicing, via spliceosome
11656	*Alas2*	*aminolevulinic acid synthase 2, erythroid*	PC00216 ^1^	Protoporphyrin-IX biosynthesis
12458	*Ccr6*	*chemokine (C-C motif) receptor 6*	GO:0006954	Inflammatory response
13211	*Dhx9*	*DEAH (Asp-Glu-Ala-His) box polypeptide 9*	GO:0050684	regulation of mRNA processing
18951	*Septin5*	*septin 5*	GO:0061640	cytoskeleton-dependent cytokinesis
66860	*Tanc1*	*tetratricopeptide repeat, ankyrin repeat and coiled-coil containing 1*	GO:0097062 ^2^	Dendritic spine maintenance
75692	*Nr2c2ap*	*nuclear receptor 2C2-associated protein*	GO:0006367 ^2^	Transcription initiation from RNA polymerase II promoter

^1^ PANTHER Protein Class ^2^ GO TERM Molecular Function.

**Table 8:** Annotation clusters with significantly enriched GO biological processes and pathways for the top 30 genes.

Category	Term	FDR ^1^	Gene symbols
GOTERM_BP	Regulation of apoptosis	0.04	*Traf1, Hgf, Bdkrb2, Tmbim6, Apaf1, Rad9a, Mapk8, Agtr1a, Mgmt, Btg1*
GOTERM_BP	Lipid localization	0.08	*Nrip1, Atp9b, Gulp1, Osbpl11*
GOTERM_BP	Negative regulation of apoptosis	0.11	*Hgf, Bdkrb2, Tmbim6, Mapk8, Agtr1a*
GOTERM_BP	Proteolysis	0.13	*Senp5, Hgf, Hectd1, Adam11, Psmb8, Apaf1, Bace1, C1qb, Ide*

^1^ FDR—false discovery rate.
